# SparkText: Biomedical Text Mining on Big Data Framework

**DOI:** 10.1371/journal.pone.0162721

**Published:** 2016-09-29

**Authors:** Zhan Ye, Ahmad P. Tafti, Karen Y. He, Kai Wang, Max M. He

**Affiliations:** 1 Biomedical Informatics Research Center, Marshfield Clinic Research Foundation, Marshfield, WI, 54449, United States of America; 2 Center for Human Genetics, Marshfield Clinic Research Foundation, Marshfield, WI, 54449, United States of America; 3 Department of Computer Science, University of Wisconsin-Milwaukee, Milwaukee, WI, 53211, United States of America; 4 Department of Epidemiology and Biostatistics, Case Western Reserve University, Cleveland, OH, 44106, United States of America; 5 Zilkha Neurogenetic Institute, University of Southern California, Los Angeles, CA, 90089, United States of America; 6 Department of Psychiatry, University of Southern California, Los Angeles, CA, 90089, United States of America; 7 Computation and Informatics in Biology and Medicine, University of Wisconsin-Madison, Madison, WI, 53706, United States of America; King Abdullah University of Science and Technology, SAUDI ARABIA

## Abstract

**Background:**

Many new biomedical research articles are published every day, accumulating rich information, such as genetic variants, genes, diseases, and treatments. Rapid yet accurate text mining on large-scale scientific literature can discover novel knowledge to better understand human diseases and to improve the quality of disease diagnosis, prevention, and treatment.

**Results:**

In this study, we designed and developed an efficient text mining framework called SparkText on a *Big Data* infrastructure, which is composed of Apache Spark data streaming and machine learning methods, combined with a Cassandra NoSQL database. To demonstrate its performance for classifying cancer types, we extracted information (e.g., breast, prostate, and lung cancers) from tens of thousands of articles downloaded from PubMed, and then employed Naïve Bayes, Support Vector Machine (SVM), and Logistic Regression to build prediction models to mine the articles. The accuracy of predicting a cancer type by SVM using the 29,437 full-text articles was 93.81%. While competing text-mining tools took more than 11 hours, SparkText mined the dataset in approximately 6 minutes.

**Conclusions:**

This study demonstrates the potential for mining large-scale scientific articles on a *Big Data* infrastructure, with real-time update from new articles published daily. SparkText can be extended to other areas of biomedical research.

## Introduction

**Fig 1:**
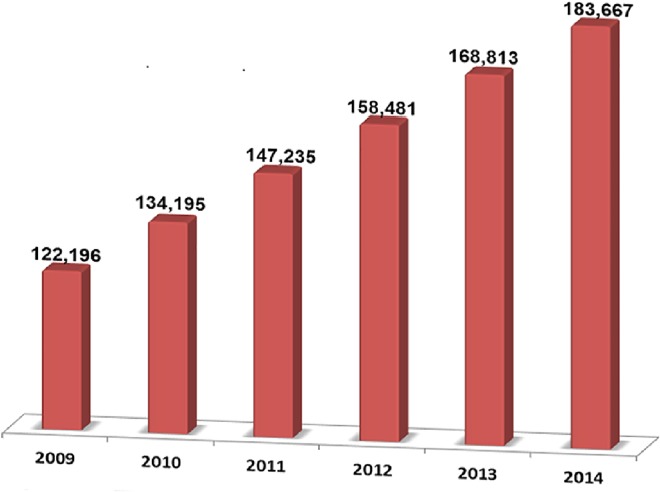
The number of publications in PubMed (http://www.ncbi.nlm.nih.gov/pubmed) over the last six years obtained by submitting a query for “cancer” in the all fields.

A large number of biomedical research articles are published every day, adding knowledge to the scientific literature on various human diseases such as cancer. As a leading cause of mortality worldwide, cancer may occur due to various causes, including internal factors such as inherited genetic mutations, hormones, and immune conditions, as well as external factors such as tobacco exposure, infectious organisms, unhealthy diet, and physical activity [1–3]. Compared to other human diseases, there have been an enormous number of scientific publications on cancer [4–6]. In the past a few years, the number of published articles in cancer research has grown consistently each year (**Fig 1**). The large amount of biomedical text data relevant to cancer studies is especially valuable for knowledge discovery related to cancer diagnosis, classification, prevention, and treatment.

Data mining can be used to discover patterns in large-scale datasets using methods at the intersection of artificial intelligence, machine learning, natural language processing (NLP), and database systems [7]. Text mining is a specialized data mining method that extracts information (e.g., facts, biological processes, diseases) from text, such as scientific literature [8,9]. Literature mining can generate new hypotheses by systematically scrutinizing large numbers of abstracts or full-text scientific articles [10,11]. Biomedical text mining and its applications have been used to improve scientific discovery in various biomedical rubrics, particularly those relevant to cancer [12–16]. Text mining strategies utilizing *Big Data* frameworks have the potential to analyze the gigantic amount of biomedical articles published in cancer research to provide operational information on cancer while providing real-time updates to incorporate newly published articles.

In this work, we investigated large-scale text mining algorithms focusing on text classification approaches for cancer research and developed an efficient and scalable framework that satisfies the following objectives: (a) to extract cancer name/type and facts about cancer research from abstracts as well as full-text articles downloaded from PubMed (http://www.ncbi.nlm.nih.gov/pubmed/); (b) to utilize and adapt NLP, text mining, and machine learning strategies in a large-scale fashion using a *Big Data* framework; and (c) to provide insights into this research area by identifying challenges and possible enhancements in large-scale biomedical text mining.

## Materials and Methods

**Fig 2:**
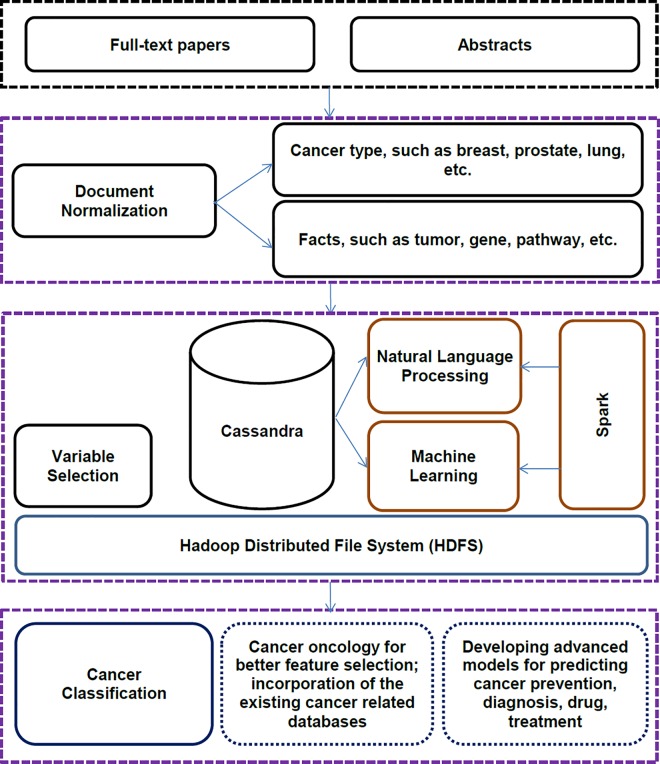
The basic framework of SparkText: We first loaded structured and unstructured abstracts and/or full-text articles into a Cassandra Database, which was then stored in multiple compute nodes. After that, we started text preprocessing and feature extraction before building prediction models based on Apache Spark. The Apache Spark Core contains the main functionalities and APIs for distributed *Big Data* solutions. As a part of Apache Spark components, the MLlib is a scalable machine learning library that includes common machine learning methods and utilities, such as classification, clustering, regression, collaborative filtering, dimensionality reduction, and underlying optimization primitives. The Standalone Scheduler allows a standalone mode cluster, which runs applications in first-in-first-out (FIFO) fashion, and each application is deployed at multiple compute nodes. The Spark Streaming Real-Time handles real-time streaming of *Big Data* files based on a micro batch style of processing.

The basic framework of SparkText is shown in **Fig 2**. It includes computational technologies, such as NLP, machine learning, *Big Data* infrastructure, and a distributed NoSQL Cassandra database system for storing the raw text information and features selected and extracted from biomedical text data. Here, we describe the design and development of SparkText to first extract disease information (e.g., breast, prostate, and lung cancer types) and then develop prediction models to classify information extracted from 19,681 abstracts and 29,437 full-text scientific articles individually.

First, we converted abstracts and/or full-text articles into a format suitable for the machine learning methods and classification tasks. A bag-of-words representation [17] using Term-Frequency–Inverse Document Frequency (TF-IDF) scores [18–20] was employed to estimate word importance for variable selection. To make the bag-of-words representation, the frequency of occurrence of each individual word or Term-Frequency (TF) is multiplied by the Inverse Document Frequency (IDF), and the TF-IDF scores are then utilized as feature vectors. The TF-IDF weighting score (*W*_*t*,*d*_) is computed by Eq (1) as follows:

Wt,d=(TFt,d)×log10⁡(NDFt) (1)

where *TF*_*t*,*d*_ refers to the frequency of the term *t* occurring in article *d*, *N* is the number of articles in the dataset, and *DF*_*t*_ refers to the number of articles containing the term *t*. *W*_*t*,*d*_ is widely used in information retrieval and text mining systems. One potential advantage of using *W*_*t*,*d*_ is the removal of irrelevant features (words). For instance, there are 1,000 articles in a dataset. Assuming the frequency of the term “almost” in the first article is 56 and the term “almost” appears in all of the 1,000 articles in the dataset, to assess the importance of the term “almost” as a feature in the dataset, the TF-IDF weighting score is calculated by the following:

W"almost",1=(56)×log10⁡(10001000)=0

As *W*_"*almost*",1_ is 0, it means that the term “almost” is not an important feature in the dataset. We used the TF-IDF weighting score to provide the bag-of-words representation as the feature vectors for both training and testing procedures. We employed three different classification algorithms, including Naïve Bayes, Support Vector Machine (SVM), and Logistic Regression, to individually build a prediction model [21–23] based on the abstracts as well as the full-text articles downloaded from PubMed. The main functionality of the prediction model is to automatically assign the abstracts/articles to one of the pre-defined categories, such as breast cancer, lung cancer, or prostate cancer. We compared the cancer categories predicted by the model with the ones classified by Medical Subject Headings (MeSH) terms [24]. We acknowledge that MeSH terms themselves are derived programmatically using more sophisticated algorithms, but we are treating them as gold standard here to evaluate whether a *Big Data* framework can reproduce predictions that match this gold standard. The proposed scalable framework was developed on a *Big Data* infrastructure, including an Apache Hadoop cluster, Apache Spark components, and a Cassandra Database. The toolset was developed in Java programming language. Use of SparkText to classify cancer types is detailed diagrammatically in **Fig 2**and further explained as follows.

### Text preprocessing

The first step of SparkText was text preprocessing in which we applied several preprocessing tasks on the raw text data (abstracts or full-text articles). This stage required a number of optional text preprocessing tasks, such as: (a) replacing special symbols and punctuation marks with blank spaces; (b) case normalization; (c) removing duplicate characters, rare words, and user-defined stop-words; and (c) word stemming. To this end, we first parsed pre-categorized (e.g., breast cancer, lung cancer, and prostate cancer by MeSH terms) abstracts or full-text articles into sentences. We then replaced special characters, such as quotation marks and other punctuation marks, with blank spaces and marked all sentences in a lower case format to provide normalized statements. Afterwards, we parsed sentences into individual words (tokens). Rare words and user-defined stop-words were removed, and the Porter Stemmer algorithm [25,26] was then used to stem all words.

### Feature extraction

**Fig 3:**
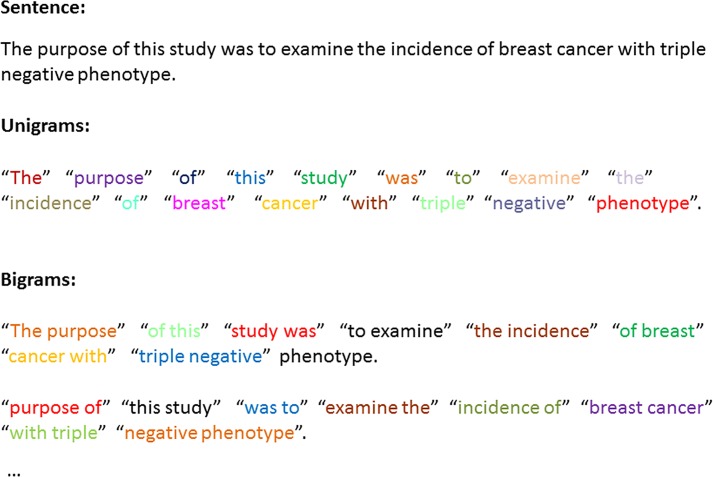
An example of unigrams and bigrams extracted from the sentence “The purpose of this study was to examine the incidence of breast cancer with triple negative phenotype. ” The sentence was chosen from an abstract downloaded from PubMed.

In regards to computational linguistics [27,28], a *N*-gram is a contiguous sequence of *N* terms from a given sentence. The *N*-gram model can be likened to putting a small window over a sentence in which only *N* words are detectable at a time. Using a 2-gram strategy, all words of a sentence are broken down into two different combinations including unigram (one word) and bigrams (two consecutive words) [29,30]. An example of these combinations is shown in **Fig 3**. We extracted a set of unigrams as well as bigrams from abstracts or full-text articles used to train SparkText for a specific cancer type. In addition to these unigrams and bigrams, the number of abstracts or full-text articles where each word appeared in the text corpus was counted. When the value of TF in an article was used as a feature value, a higher weight was assigned to words that appeared frequently in a corpus. Hence, the IDF was a much better value, since it assigned a lower weight to frequent terms. We calculated IDF as the log ratio of the number of abstracts or full-text articles in the training set to the number of abstract or full-text articles containing the term. Combining these numbers as a TF/IDF weighting is the best known weighting scheme in text retrieval [30]. This weighting scheme was completely valid not only for unigrams but also for bigrams, trigrams, and others. During the performance of these tasks, we converted all abstracts or full-text articles into “equal-length” numeric feature vectors where every feature presented the TF-IDF of a unigram and/or bigrams in a full-text article or abstract instance. While using unigrams and/or bigrams in different datasets including abstract and/or full-text, there were thousands to tens of thousands of features. All abstracts or full-text articles along with their feature vectors were organized in a bag-of-words representation model. A brief example of a bag-of-words representation is shown in **Table 1**.

**Table 1:** An example of a bag-of-words representation. The terms “biology”, “biopsy”, “biolab”, “biotin”, and “almost” are unigrams, but “cancer-surviv”, and “cancer-stage” are bigrams. Using TF/IDF weighting scores, the feature value of the term “almost” equals to zero.

Article ID	biolog	biopsi	biolab	biotin	almost	cancer-surviv	cancer-stage	Article Class
00001	12	1	2	10	0	1	4	breast-cancer
00002	10	1	0	3	0	6	1	breast-cancer
00014	4	1	1	1	0	28	0	breast-cancer
00063	4	0	0	0	0	18	7	breast-cancer
00319	0	1	0	9	0	20	1	breast-cancer
00847	7	2	0	14	0	11	5	breast-cancer
03042	3	1	3	1	0	19	8	lung-cancer
05267	4	4	2	6	0	14	11	lung-cancer
05970	8	0	4	9	0	9	17	lung-cancer
30261	1	0	0	11	0	21	1	prostate-cancer
41191	9	0	5	14	0	11	1	prostate-cancer
52038	6	1	1	17	0	19	0	prostate-cancer
73851	1	1	8	17	0	17	3	prostate-cancer

### Training and evaluating prediction models

Completion of the above steps resulted in conversion of all abstracts or full-text articles into a representation module suitable for the machine learning methods. We applied three well-known classification methods, namely Naïve Bayes, SVM, and Logistic Regression, to train and build prediction models. We utilized the scalable Apache Spark MLlib classification components, such as SVM, Logistic Regression, and Naïve Bayes classifiers that have been originally developed by the Apache Foundation. In a classification problem, data are labeled by being assigned to a class or category (e.g., “Breast Cancer” or “Lung Cancer”). Then the decisions modeled are to assign labels to new unlabeled data. This can be thought of as a discrimination problem, modeling the similarities or differences between groups. Many classification algorithms can be formulated as a convex optimization problem, i.e., the task of finding a minimizer of a convex function f that is associated with a variable vector w which has d number of entries. We can briefly describe this expression as the optimization problem:

$$\min f(w)\;w\in R^{d}$$ (2)

where the objective function is as follows:

$$f(w)≔a\;R(w)+\frac{1}{n}\;\sum_{i=1}^{n}L(w;\;x(i),y(i))$$ (3)

where the vectors *x*(*i*) ∈ R^d^ are the training instances (1≤ i≤ n), and y(i) ∈ R are theirs labels (classes), which we would like to predict. We will call the method linear if L(w; x,y) could be express as a function of w^T^x and y. The objective function f has two parts: the regularizer (R(w)) that take cares of the complexity of the model, and the loss that measures the error of the model on the training instances. The loss function L(w;.) is formally a convex function in w. The fixed regularization parameter a ≥ 0 defines the trade-off between the two objectives: (a) minimizing the loss (i.e., training error), and (b) minimizing model complexity (i.e., to avoid the problem of overfitting in which we will have small training error and large testing error). Most of the Apache Spark classification components fall into this model. The Apache Spark SVM component utilizes linear kernel and it can be trained with L1 (Eq (4)) and L2 regularizations (Eq (5)) [31]. By default, the Apache Spark SVM component uses L2 regularization as Eq (5).

$${\left\|w\right\|}_{1}$$ (4)

The Apache Spark SVM component can provide L1 regularization (30) as Eq (4):

$$\frac{1}{2}\;{\left\|w\right\|}_{2}^{2}$$ (5)

Apache Spark Logistic Regression component also offers a linear kernel based on Eq (3) along with a loss function given by the following expression:

$$L(w;\;x,y)=\log\left(1+\exp\left(-yw^{T}x\right)\right)$$ (6)

For a binary classification problem, the component outputs a binary logistic regression model. Given a new data point, denoted by x, this model will make predictions using a logistic function as Eq (7):

$$f(z)=\frac{1}{1+e^{-z}}$$ (7)

where z = w^T^x. By default, if w^T^x > 0.5, the outcome would be positive; otherwise, it would be negative. By default, the first class 0 is chosen as the “pivot” class. For Logistic Regression, the L2 regularization (Eq (5)) was employed to control overfitting with a large amount of features in the model building process [32], but it can support all three possible regularizations (none, L1, or L2). For the Naïve Bayes model, the only assumption was that every pair of features is independent as recommended for documentation classification by Apache Spark [33]. In the proposed SparkText framework, we utilized the Apache Spark MLlib classification components including SVM, Logistic Regression, and Naïve Bayes using their default parameters. To better experimentally validate the proposed framework, we examine their attributes using both default and non-default parameters as illustrated in the results. In our evaluation, ten 5-fold cross-validation experiments were performed to assess the model performance using both abstracts and full-text articles. For each of the experiments, the dataset was partitioned into five equal-sized subsamples by random split. Of the five subsamples, one was retained as the testing dataset while the rest were used for model building as the training dataset. The cross-validation process was repeated five times. Then the average accuracy of predictions across all experiments was computed.

### *Big Data* processing using Apache Spark component and Cassandra database

Biomedical text mining can generate new hypotheses by systematically examining a huge number of abstracts and/or full-text articles of scientific publications. With the use of large-scale data published in biomedical literature, a key challenge is appropriate management, storage, and retrieval of high volume data. When the data volume is in the terabyte range, the data must be segmented them into chunks of manageable size in order to be retrieved and analyzed on distributed computation frameworks. To tackle the challenges of large-scale text classification, we developed the proposed toolset using Apache Spark (http://spark.apache.org/) and Apache Cassandra database (http://cassandra.apache.org/). Apache Spark is an open source *Big Data* processing framework built around speed, performance, scalability, reusability, and sophisticated analytics. It provides simple and expressive programming models that support a wide range of applications, including ETL (Extract, Transform and Load), machine learning, stream processing, and graph computation. It is also a scalable framework that provides a high-level application programming interface (API) and a consistent architect model for *Big Data* management. Spark was originally developed in the AMPLab at the University of California Berkeley in 2009, offering more than 80 high-level operators to make parallel applications. Cassandra database is an open source distributed database system that handles large amounts of data. SparkText supports scalability and high availability with no single point of failure (**Fig 2**).

## Results

To evaluate the performance, accuracy, and running time of SparkText, extensive experiments were performed on the abstracts and full-text articles downloaded from PubMed. In the sections below, we first describe the testing dataset and the experimental setup, then report measurement and comparison of the accuracy of SparkText using different machine learning methods on three different datasets. We have utilized the Apache Spark MLlib components using their default parameter values. To further examine the SparkText attributes, we also analyze the accuracy of the SparkText using non-default Apache Spark MLlib parameter values. After that, we report comparison of the accuracy and runtime efficiency of SparkText with two open source toolsets, Weka Library [34,35] and TagHelper Tools[36].

### Experimental setup

We downloaded abstracts and full-text articles from PubMed (**Datasets in S1 File**) to generate both training and testing datasets. Datasets and their attributes are shown in **Table 2**. Separating a dataset into “training data” and “testing data” is an important part of evaluating text classification models. In such a dataset, a training set is used to build up a prediction model, while a testing set is used to evaluate the model built. To this end, for each dataset illustrated in **Table 2**, we employed 5-fold cross validation, each time using 80% of the entities to train a prediction model and the remaining 20% to test it. We utilized 64-bit Linux CentOS operating system on a cluster platform built with 20 data nodes, each configured with 6 GB memory, two CPUs (2.6 GHz), and 1 TB of hard disk space.

**Table 2:** The datasets: all abstracts and full-text articles were downloaded from PubMed. The datasets included abstracts and full-text articles related to three types of cancer, including breast, lung, and prostate cancer. For each dataset, we employed 80% of the entire dataset to train a prediction model while the remaining 20% was used for testing.

Dataset	Year Range	# Instances	# Breast Cancer	# Lung Cancer	# Prostate Cancer
Abstracts	2011–2016	19,681	6,137	6,680	6,864
Full-text Articles I	2011–2016	12,902	4,319	4,281	4,302
Full-text Articles II	2009–2016	29,437	9,787	9,861	9,789

### Accuracy validation

**Fig 4:**
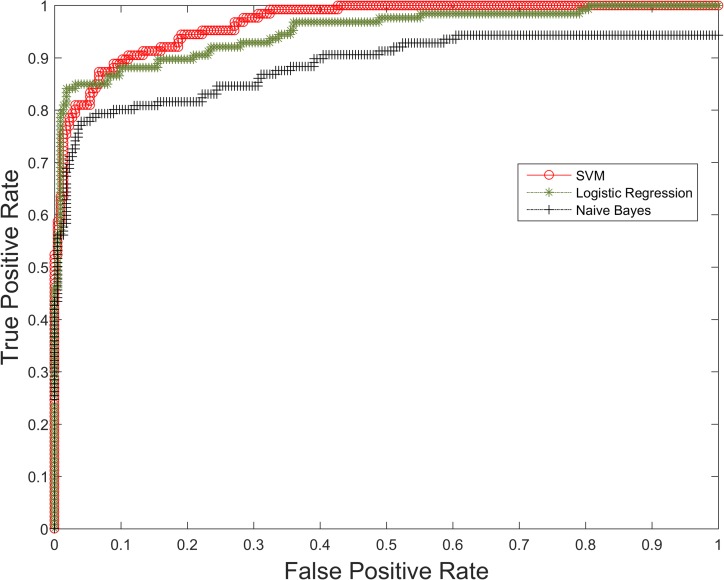
The ROC curves for the dataset “Full-text Articles II”: the area under the curve for the SVM classifier represents a better result compare to that of the Naïve Bayes and Logistic Regression algorithms.

We assessed the accuracy of the proposed prediction models using three common measures including accuracy, precision, and recall. Accuracy describes the percent of predictions that are correct, precision (also called positive predictive value) refers to the percent of positive predictions that are correct, and recall (also called sensitivity) describes the percent of positive cases that are detected [21,22]. Given *P* positive instances and *N* negative instances in an experiment, the four potential outcomes include true positive (*TP*), true negative (*TN*), false positive (*FP*), and false negative (*FN*). Then accuracy (*ACC*) = (*TP* + *TN*)/(*P* + *N*), precision (*PPV*) = *TP*/(*TP* + *FP*), and recall (true positive rate) (*TPR*) = *TP*/(*TP* + *FN*). **Table 3**shows quantitative results for accuracy, precision, and recall of SparkText. In this experiment, we employed three classification methods on the three different datasets illustrated in **Table 2**. Results in **Table 3**were obtained by utilizing only default parameter values of the Apache Spark MLlib components including SVM, Logistic Regression, and Naïve Bayes classifiers. Experimental results using both default and non-default parameter values of the Apache Spark MLlib classifiers are presented in **Table 4**. We also assessed the Receiver Operating Characteristic (ROC) curves [22,23] for quantitative comparison of the prediction models using the three different machine learning methods. **Fig 4**shows representative ROC curves generated in a typical experiment using the “Full-text Articles II” data. The area under the curve for the SVM classifier represented a reasonable test, while the area for the Naïve Bayes classifier compared poorly to the two other classification methods. The accuracy of SVM classifier on the dataset was better than Naïve Bayes or Logistic Regression.

**Table 3:** The quantitative results for accuracy, precision, and recall of SparkText using three datasets. For each dataset, 80% was used to train a prediction model and the remaining 20% for testing.

Dataset	Classifier	Accuracy	Precision	Recall
Abstracts	SVM	94.63%	93.11%	94.81%
Abstracts	Logistic Regression	92.19%	91.07%	89.49%
Abstracts	Naïve Byes	89.38%	89.13%	90.82%
Full-text Articles I	SVM	94.47%	92.97%	93.14%
Full-text Articles I	Logistic Regression	91.05%	90.77%	89.19%
Full-text Articles I	Naïve Bayes	88.02%	89.01%	90.68%
Full-text Articles II	SVM	93.81%	91.88%	92.27%
Full-text Articles II	Logistic Regression	90.57%	90.28%	91.59%
Full-text Articles II	Naïve Bayes	86.44%	87.61%	89.12%

**Table 4:** The quantitative results for accuracy using different regularization parameters. For each dataset, 80% was used to train a prediction model and the remaining 20% for testing.

Classifier	Dataset	Regularization Parameter	Accuracy
SVM	Abstracts	L2 (Default)	94.63%
SVM	Abstracts	L1	91.07%
SVM	Abstracts	None	89.72%
Logistic Regression	Abstracts	L2 (Default)	92.19%
Logistic Regression	Abstracts	L1	90.61%
Logistic Regression	Abstracts	None	88.54%
SVM	Full-text Articles I	L2 (Default)	94.47%
SVM	Full-text Articles I	L1	90.33%
SVM	Full-text Articles I	None	88.51%
Logistic Regression	Full-text Articles I	L2 (Default)	91.05%
Logistic Regression	Full-text Articles I	L1	88.19%
Logistic Regression	Full-text Articles I	None	87.04%
SVM	Full-text Articles II	L2 (Default)	93.81%
SVM	Full-text Articles II	L1	90.16%
SVM	Full-text Articles II	None	87.94%
Logistic Regression	Full-text Articles II	L2 (Default)	90.57%
Logistic Regression	Full-text Articles II	L1	87.63%
Logistic Regression	Full-text Articles II	None	86.71%

Comparing the proposed methods using abstracts and full-text articles, we found that the accuracy of the prediction models using abstracts was better than that of full-text articles. This is somewhat counter-intuitive, but a possible explanation might be related to the size of the feature space, which seems to be too large for full-text articles. For text classification purposes, abstracts may work better than full-text scientific articles. However, to tackle the problem of information retrieval and knowledge discovery, full-text articles are expected to provide a richer source of information compared to abstracts alone. Therefore, future work will attempt to take feature reduction strategies into account to improve prediction models based on full-text articles.

### Quantitative comparisons of the prediction models

**Fig 5:**
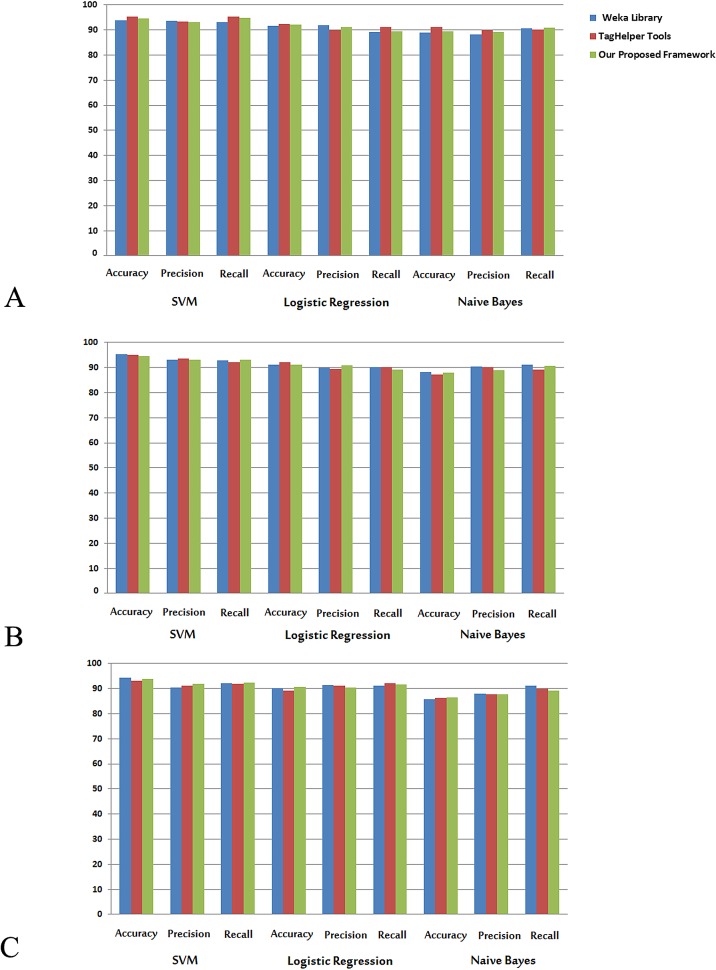
**Quantitative comparisons of the prediction models on text mining**: (A) the accuracy, precision, and recall obtained from 19,681 abstracts; (B) the accuracy, precision, and recall on 12,902 full-text articles; and (C) the accuracy, precision, and recall on 29,437 full-text articles. Table 2 provides the details on these 3 datasets. Five-fold cross validation was used in all analyses.

We also compared the accuracy, precision, and recall of SparkText with two open source toolsets, Weka Library and TagHelper Tools. **Fig 5**shows quantitative comparisons of the prediction models using Naïve Bayes, SVM, and Logistic Regression on different datasets including abstracts and full-text articles. This experiment illustrates that the difference between accuracy estimated by the SparkText framework and accuracy estimated by two well-known Weka Library and TagHelper Tools is less than 1%. Therefore, the SparkText framework generates reasonable results regarding the accuracy of the prediction models. The accuracy of SparkText is promising, as Weka Library and Tag Helper tools have been in use for several years to solve the problems of text data classification. In addition to having comparable accuracy, precision, and recall as the two widely used text mining libraries, SparkText showed much better performance as described below.

### Performance comparison

Comparisons of running time among SparkText and the Weka Library and Tag Helper tools are shown in **Table 5**. Using the dataset “Abstracts”, SparkText took approximately 3 minutes to complete classification, while the Weka Library and TagHelper Tools took approximately 138 minutes and 201 minutes, respectively. Employing the dataset “Full-text Articles I”, SparkText took approximately 4 minutes to complete classification, while the Weka Library and TagHelper Tools took almost 309 minutes and 571 minutes, respectively. On the dataset “Full-text Articles II”, SparkText took approximately 6 minutes to complete classification, while Weka Library and TagHelper Tools both took more than 11 hours. The speed advantage of SparkText becomes more apparent as the input dataset becomes larger. For the largest dataset, which included 29,437 full-text articles, the proposed scalable framework achieved a speed 132 times faster than that of commonly used text mining tools including Weka Library and TagHelper Tools (**Table 5**).

**Table 5:** Comparing the time efficiency results, SparkText outperformed other available text mining tools with speeds up to 132 times faster on the larger dataset that included 29,437 full-text articles.

Tools	Dataset	≅ Running Time (minutes)
Weka Library	Abstracts	138
Tag Helper Tools	Abstracts	201
**SparkText**	**Abstracts**	**3**
Weka Library	Full-text Articles I	309
Tag Helper Tools	Full-text Articles I	571
**SparkText**	**Full-text Articles I**	**4**
Weka Library	Full-text Articles II	697
Tag Helper Tools	Full-text Articles II	768
**SparkText**	**Full-text Articles II**	**6**

## Discussion

This work focused on investigating large-scale biomedical text classification for massive datasets downloaded from PubMed. We utilized NLP, machine learning strategies, and *Big Data* infrastructure to design and develop a distributed and scalable framework to extract information, such as cancer type for breast, prostate, and/or lung cancers, and then to develop prediction models to classify information extracted from tens of thousands of abstracts and/or full-text articles downloaded from PubMed by associated MeSH terms. The SparkText framework was developed on a *Big Data* infrastructure, including an Apache Hadoop cluster, together with an Apache Spark component and Cassandra database. The accuracy of predicting a cancer type by SVM using the abstracts was 94.63%, while its accuracy using the 29,437 full-text articles (Full-text II) was 93.81%. The developed toolset was more than 130 times faster than other existing methods for mining a large dataset, which included 29,437 full-text articles. This demonstrates the potential of mining large-scale scientific articles on a *Big Data* infrastructure. The time efficiency and accuracy of SparkText are both promising, and this strategy will provide tangible benefits to biomedical research. The package of the developed toolsets in this study is freely available for use by academic or non-profit organizations at http://sparktext.omicspace.org/. Source code of the package can be downloaded after obtaining a license agreement from Marshfield Clinic Applied Sciences.

This pilot study only leveraged three of the available machine learning methods implemented in Apache Spark component and tens of thousands of articles downloaded from PubMed to classify three cancer types. In this work, we did not analyze whether an article focused on single cancer or multiple cancers (**Overlapping of different cancer types in S1 File**). In future studies, we plan to employ larger datasets, including hundreds of thousands full-text articles, to assess the accuracy, scalability, and runtime efficiency of SparkText. We will focus on feature extraction and dimension reduction to provide noteworthy features; hence, condensing the feature space for text classification methods. To provide better knowledge and information to cancer research, we intend to work on multi-dimensional classification tasks to classify information extracted from scientific articles not only on specific cancer types, but also on cancer treatment, diagnosis, and prevention categories. Furthermore, besides utilizing the available machine learning methods implemented in Apache Spark component and available published articles on PubMed, we also plan to develop novel machine learning approaches for discovering the associations between gene and disease/phenotype, gene and drug dosage/use, and other associations to advance precision medicine.

## Conclusions

This study demonstrates the potential for mining large-scale scientific articles on a *Big Data* infrastructure, with real-time update from new articles published daily. SparkText can be extended to other areas of biomedical research.

## Supporting Information

S1 File (DOCX)
