# Supplementary material for: SparkText: Biomedical Text Mining on Big Data Framework
Source: PLoS One. 2016 Sep 29;11(9):e0162721. doi: 10.1371/journal.pone.0162721 (PMC5042555; doi:10.1371/journal.pone.0162721)
Supplement: S1 File — (DOCX) [file pone.0162721.s001.docx]

**S1 File**

**SparkText: biomedical text mining on big data framework**

**Datasets:**

All the abstracts and full text articles are extracted from the following link: [***https://www.ncbi.nlm.nih.gov/pubmed/advanced***](https://www.ncbi.nlm.nih.gov/pubmed/advanced)

The **queries** used to create the datasets are as follows:

For **Breast Cancer** data, we used *(((((((((((breast cancer[MeSH Major Topic]) AND breast cancer[Title]) AND ("2011/01/01"[Date - MeSH] : "2016/02/25"[Date - MeSH]))))) AND English[Language])))))*, then all the abstracts and full text articles within this search considered will have the label as breast cancer for our further experiment.

For **Prostate Cancer** data, we used *((((((((((((prostate cancer[MeSH Major Topic]) AND prostate cancer[Title]) AND ("2011/01/01"[Date - MeSH] : "2016/02/25"[Date - MeSH]))))) AND English[Language]))))))*, then all the abstracts and full text articles within this search considered will have the label as prostate cancer for our further experiment.

For **Lung Cancer** data, we used *((((((((((((((lung cancer[MeSH Major Topic]) AND lung cancer[Title/Abstract]) AND ("2011/01/01"[Date - MeSH] : "2016/02/25"[Date - MeSH]))))) AND English[Language]))))))))*, then all the abstracts and full text articles within this search considered will have the label as lung cancer for our further experiment.

**Overlapping of different cancer types described in the same literature:**

For the currently analyses in this manuscript, we did not analyze whether the study focused on single cancer or multiple cancers. To generate the datasets for this study, we used the Medical Subject Headings (MeSH) (https://www.nlm.nih.gov/mesh/) system, which aims to catalog large scale biomedical text classification. After further investigation, we have counted the number of overlaps for every single class (e.g., breast cancer) in three different datasets, which is presented in the Table below.

| Dataset: **Abstracts** | |
| --- | --- |
| Total number of articles: **19,681** | |
| Number of articles discussed **Breast Cancer**: **6,137**  Number of **overlaps** with Prostate Cancer:**141**  Number of **overlaps** with Lung Cancer: **187** | **Overlaps Ratio: 2.29%**  **Overlaps Ratio: 3.04%** |
| Number of articles discussed **Lung Cancer**: **6,680**  Number of **overlaps** with Breast Cancer: **186**  Number of **overlaps** with Prostate Cancer: **223** | **Overlaps Ratio: 2.78%**  **Overlaps Ratio: 3.33%** |
| Number of articles discussed **Prostate Cancer**: **6,864**  Number of **overlaps** with Lung Cancer: **185**  Number of **overlaps** with Breast Cancer:**161** | **Overlaps Ratio: 2.69%**  **Overlaps Ratio: 2.34%** |
|  | |
| Dataset: **Full Text Articles I** | |
| Total number of articles: **12,902** | |
| Number of articles discussed **Breast Cancer**: **4,319**  Number of **overlaps** with Lung Cancer: **94**  Number of **overlaps** with Prostate Cancer:**112** | **Overlaps Ratio: 2.17%**  **Overlaps Ratio: 2.59%** |
| Number of articles discussed **Lung Cancer**: **4,281**  Number of **overlaps** with Prostate Cancer: **92**  Number of **overlaps** with Breast Cancer: **91** | **Overlaps Ratio: 2.14%**  **Overlaps Ratio: 2.12%** |
| Number of articles discussed **Prostate Cancer**: **4,302**  Number of **overlaps** with Breast Cancer: **95**  Number of **overlaps** with Lung Cancer: **119** | **Overlaps Ratio: 2.20%**  **Overlaps Ratio: 2.76%** |
|  | |
| Dataset: **Full Text Articles II** | |
| Total number of articles: **29,437** | |
| Number of articles discussed **Breast Cancer**: **9,787**  Number of **overlaps** with Prostate Cancer: **328**  Number of **overlaps** with Lung Cancer: **293** | **Overlaps Ratio: 3.35%**  **Overlaps Ratio: 2.99%** |
| Number of articles discussed **Lung Cancer**: **9,861**  Number of **overlaps** with Breast Cancer:**302**  Number of **overlaps** with Prostate Cancer: **281** | **Overlaps Ratio: 3.06%**  **Overlaps Ratio: 2.84%** |
| Number of articles discussed Prostate Cancer: **9,789**  Number of **overlaps** with Breast Cancer: **286**  Number of **overlaps** with Lung Cancer: **311** | **Overlaps Ratio: 2.92%**  **Overlaps Ratio: 3.17%** |

As described in the Table above, the average ratio of having overlaps in the first, second, and third dataset are calculated as **2.74%**, **2.33%**, and **3.05%** respectively. When the overlaps ratio is high, the remedy can be sought in utilizing neural networks, deep learning algorithms, or/and schemas for finding overlapping regions. They can be more flexible in the way that a “separation” (whether linear or not) is learned in the hidden layers without requiring from us to decide on a kernel. Since the average ratio of overlaps in the obtained datasets of our study is not high, we have not delved into those strategies in this study. We will take deep learning strategies into account as part of our future work.
